# Large solitary luteinized follicle cyst of pregnancy and puerperium: report of two cases

**DOI:** 10.1186/1746-1596-6-3

**Published:** 2011-01-10

**Authors:** Michele Lomme, Stefan Kostadinov, Cunxian Zhang

**Affiliations:** 1 Department of Pathology and Laboratory Medicine, Women & Infants Hospital of Rhode Island, Warren Alpert Medical School of Brown University, Providence, USA

## Abstract

We describe two cases of large solitary luteinized follicle cyst of pregnancy and puerperium (LSLFCPP) with new clinicopathologic findings. The first case occurred in a 40-year old woman who was found to have a left ovarian mass during the third trimester of pregnancy. The patient delivered a full term healthy female infant via caesarean section. The ovarian mass was removed by oophorectomy. The specimen showed a unilocular, thin-walled, clear fluid filled cyst measuring 15 × 12 × 5 cm. Microscopically, the cyst was lined by single to multiple layers of luteinized cells with mainly small, round and regular nuclei and focally enlarged, bizarre, and hyperchromatic nuclei. Occasional mitotic figures were seen. The cyst wall showed marked edema and nests of luteinized cells that were morphologically similar to the cyst lining cells. Groups of lesional cells were surrounded by reticulin fibers. The patient has been healthy without disease after 7 years. The second patient was a 29-year old pregnant woman who was found to have a right ovarian cyst by ultrasound at 14-week gestation. She then presented with preterm labor at 33-week gestation and delivered a healthy female infant via caesarean section. A right salpingo-oophorectomy was performed. Gross inspection of the specimen revealed a unilocular, brown mucoid fluid filled cyst measuring 14 × 11 × 9 cm. The cyst surfaces were smooth, and the cyst wall exhibited marked edema. Microscopic examination showed features similar to the first case: cyst lined by luteinized cells with focal large nuclei, scattered nests of luteinized cells in the edematous fibrous wall, and reticulin fibers surrounding large nests of lesional cells. No mitoses, however, were identified in the second case. The patient has been well without disease 1 year after surgery. These two cases contribute to a better understanding of LSLFCPP. Our case in the 40-year old patient is the first to show mitotic figures in LSLFCPP and suggests that the presence of occasional mitoses should not exclude a diagnosis of LSLFCPP. The lesion in the second patient caused preterm labor. Nevertheless, absence of disease recurrence in our patients demonstrates a benign nature of LSLFCPP.

## Background

Ovarian tumors and tumor-like masses during pregnancy are uncommon, with an incidence of about 1% in one large study consisting of 8420 patients (1). Most neoplasms are benign, and about 4% are malignant (1). Tumor-like lesions include pregnancy luteoma, hyperreactio luteinalis, intrafollicular granulosa cell proliferation, hilus cell hyperplasia, ectopic decidua, and large solitary luteinized follicle cyst of pregnancy and puerperium (LSLFCPP) (1-2). These lesions can simulate neoplasms by clinical, gross, and microscopic examinations. Of particular interest is LSLFCPP for its enormous size and confusion with neoplasms. LSLFCPP is a rare lesion; only about 10 cases have been reported in the literature. We now describe the clinicopathologic features of LSLFCPP in two patients.

## Case presentations

### Case1

This was a 40 year old, premigravida patient who initially presented to the infertility clinic at our institution for desired pregnancy. Her past medical history was significant for primary infertility. A successful intrauterine pregnancy was achieved via intrauterine insemination. Due to advanced maternal age, the patient underwent extensive prenatal testing and monitoring through the course of her pregnancy. All tests were normal, with the exception of a left ovarian mass that was incidentally detected by ultrasound during the third trimester. The patient was closely followed without prenatal surgical intervention. Her pregnancy advanced uneventfully, and labor commenced at 40-week gestation. Due to failure to progress, a caesarean section was performed resulting in the delivery of a healthy female infant. At the time of caesarean section, a left oophorectomy was performed.

The specimen was received fresh for intraoperative pathology consultation. On gross examination, it consisted of an intact, unilocular, thin-walled cyst measuring 15 × 12 × 5 cm and filled with clear fluid. Both the outer and the inner surfaces of the cyst were smooth. The cyst wall ranged from 0.1 cm to 0.8 cm in thickness and showed marked edema. Based on the gross findings, an intraoperative interpretation of benign ovarian cyst was made. No frozen section was performed.

**Figure 1:**
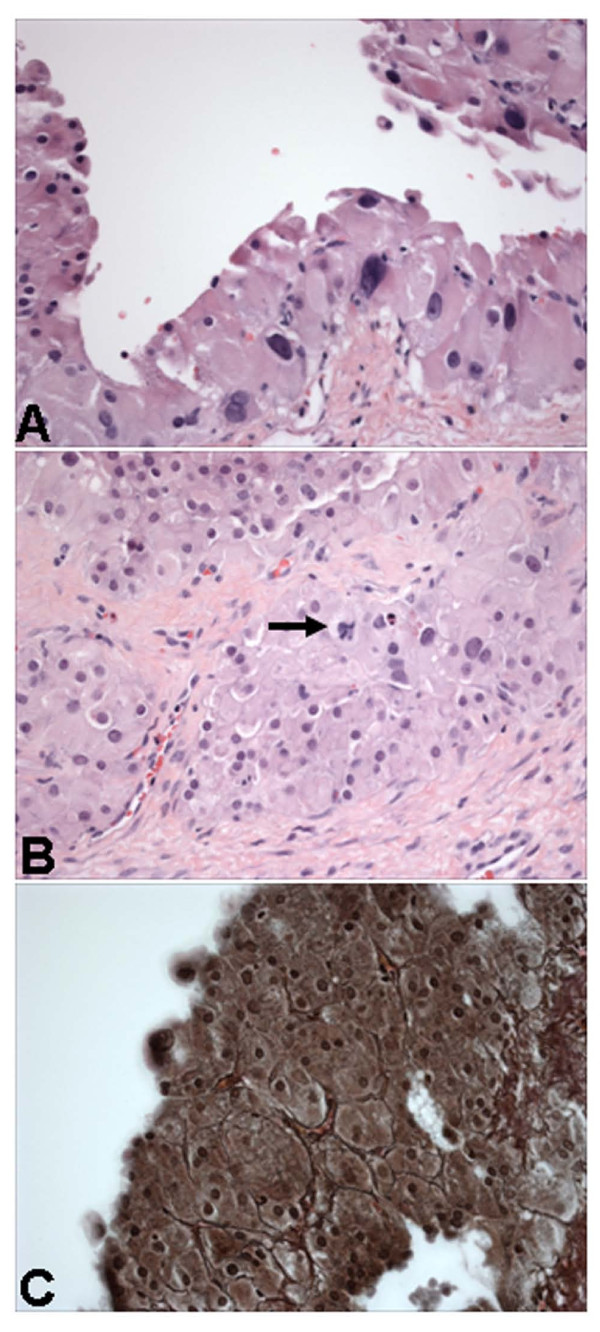
**Microscopic features of ovarian cyst in case 1**. The cyst is lined by several layers of luteinized cells showing focal marked nuclear pleomorphism (A) and occasional mitotic figures (B). Reticulin fibers surround nests of lesional cells (C). Hematoxylin and eosin stain in A and B, and reticulin stain in C. Magnifications: ×400 in A, B, and C.

On subsequent microscopic examination, the cyst was lined by single to multiple layers of large cells with abundant eosinophilic cytoplasm (Figure 1A). Most cells showed small, round and regular nuclei, but focal cells displayed enlarged and bizarre nuclei with hyperchromasia and occasional mitosis (Figure 1B). The outer fibrous wall of the cyst showed edema and nests of luteinized cells that were morphologically similar to the cyst lining cells. Special stain showed reticulin fibers around nests of luteinized cells in the cyst lining (Figure 1C) and in the outer cyst wall. Adjacent residual ovarian tissue exhibited a corpus luteum and numerous cystic follicles.

The patient has been free from disease for 7 years after surgery.

### Case 2

The patient was a 29-year old pregnant woman who previously delivered through caesarean sections two healthy full term infants. Her past medical history was significant for gastroesophageal reflux disease, asthma, obesity, gallstones, and smoking. Ultrasound at 14-week gestation revealed a right ovarian cyst. At 33-week gestation, the patient presented with preterm labor. With repeat caesarean section, a healthy female infant was delivered. At the time of caesarean section, a right salpingo-oophorectomy was performed after approximately 2 liters of fluid were drained from the ovarian cyst.

We received the specimen for intraoperative consultation. Gross inspection revealed a cystic lesion measuring 14 × 11 × 9 cm. The external surface was maroon and smooth with prominent vascular markings. Sectioning showed a unilocular cyst filled with brownish mucoid fluid. The cyst wall measured from 0.5 cm to 1.0 cm in thickness and showed severe edema. The inner surface of the cyst was flat and displayed scattered patches of hemorrhage but no solid or papillary tumor was identified. Attached to the cyst was a residual normal appearing ovary measuring 3 × 2.5 × 1.5 cm and an unremarkable segment of fallopian tube measuring 3 × 0.5 cm. The gross findings indicated a benign ovarian cyst, and thus no frozen section was performed.

**Figure 2:**
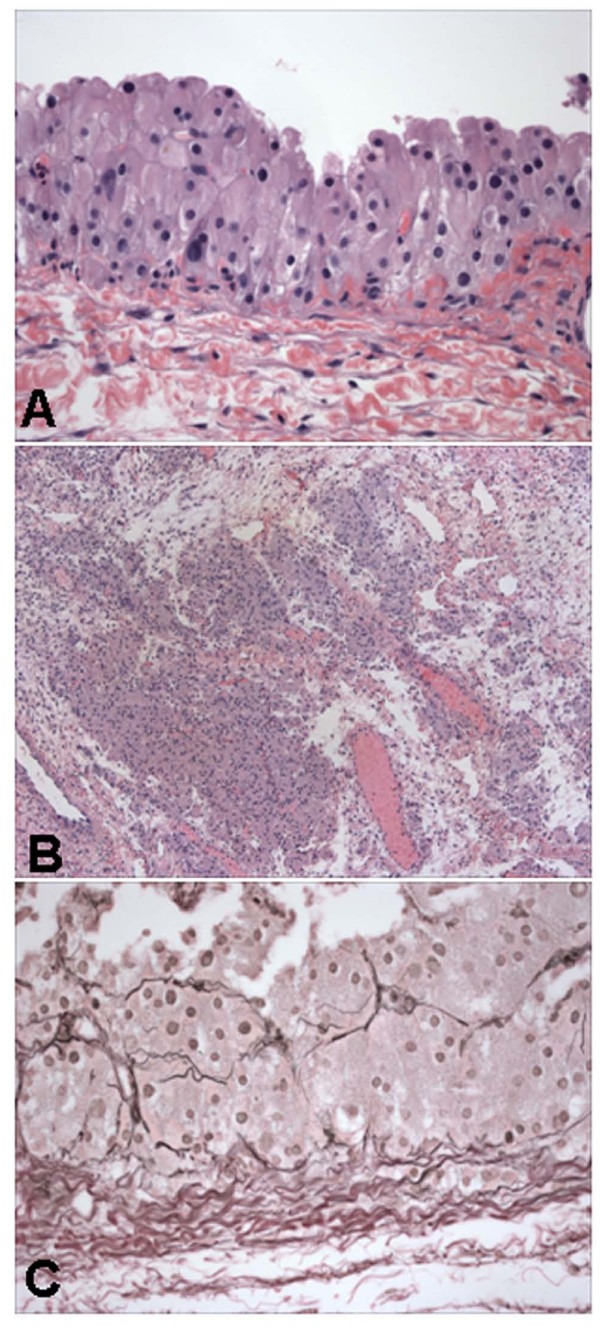
**Histologic characteristics of ovarian cyst in case 2**. The cyst is lined by multiple layers of luteinized cells showing focal nuclear pleomorphism (A). The outer cyst wall shows nests of luteinized cells (B). Nests of lesional cells are surrounded by reticulin fibers (C). Hematoxylin and eosin stain in A and B, and reticulin stain in C. Magnifications: ×400x in A and C, and ×100 in B.

On microscopic examination, the cyst was lined by one to several layers of luteinized cells that exhibited abundant eosinophilic cytoplasm with focal vacuolization. Most nuclei were small and round but focal nuclei were irregular and enlarged (Figure 2A). No mitosis was identified. The outer cyst wall consisted of edematous fibrous tissue and scattered nests of luteinized cells that morphologically resembled the cyst lining cells (Figure 2B). Special stain showed reticulin fibers surrounding large nests of luteinized cells in the cyst lining (Figure 2C) and in the outer cyst wall. Remnant ovarian tissue showed a corpus luteum and multiple cystic follicles. The segment of fallopian tube was unremarkable.

The patient has been well without disease after 1 year since surgery.

## Discussions

Our two cases of LSLFCPP demonstrated many clinicopathologic features similar to those described in the literature (3-6), including a unilateral, unilocular cyst and the presence of focal bizarre nuclei. However, our case in the 40-year old patient was the first to show mitotic figures. This finding indicates that the presence of focal mitoses should not exclude a diagnosis of LSLFCPP. Although most patients with LSLFCPP do not show complications (3), our second patient experienced preterm labor at 33-week gestation. As examination of placenta and review of prenatal history did not show other causes of preterm labor in this patient who previously delivered two infants at full term, LSLFCPP was most likely the culprit of the patient's preterm labor. Our cases demonstrate that LSLFCPP is benign as the patients have been free of disease since surgery for 7 and 1 year, respectively.

The pathogenesis of LSLFCPP is unclear. Its occurrence during pregnancy suggests a role of human chorionic gonadotropin (hCG). This association is supported by the presence of numerous cystic follicles, which are known to be induced by hCG, in the remnant ovaries of our cases. The literature, however, has described several cases of LSLFCPP occurring late in the puerperium when hCG levels are low (3). Thus hCG may not be the only contributing factor. Although lined by luteinized cells, the cyst is unlikely to originate from a corpus luteum since a separate corpus luteum was seen in the remnant ovaries of our cases and in the contralateral ovary of another case (3). The presence of reticulin fibers around groups of lesional cells supports a granulosa cell origin from a follicle.

The major differential diagnoses include the unilocular cystic granulosa cell tumors of both the adult and the juvenile types. Although LSLFCPP is indistinguishable grossly from unilocular cystic granulosa cell tumor, they differ in microscropic features. Unlike LSLFCPP that show luteinized cells with focal bizarre nuclei, adult granulosa cell tumor is composed of a monotonous population of granulosa cells usually without luteinization and bizarre nuclei (7). Furthermore, nuclear grooves and Call-Exner bodies are seen in granulosa cell tumor but not in LSLFCPP. Juvenile granulosa cell tumor may create a greater diagnostic problem as the neoplastic cells often exhibit abundant eosinophilic cytoplasm and occasional bizarre nuclei. However, juvenile granulosa cell tumor is mitotically active while LSLFCPP shows absent to occasional mitotic figures.

## Conclusions

The findings in our two cases contribute to a better understanding of LSLFCPP. Our case in the 40-year old patient is the first to show mitoses in LSLFCPP and indicates that the presence of occasional mitoses should not exclude the diagnosis of LSLFCPP. The lesion in the second patient caused preterm labor. Nevertheless, absence of disease recurrence in our patients demonstrates that LSLFCPP is benign.

## Competing interests

The authors declare that they have no competing interests.

## Authors' contributions

ML collected case information and participated in manuscript writing. SK collected case information and reviewed manuscript. CZ collected case information and wrote the manuscript. All authors read and approved the final manuscript.
